# DNA barcode markers for birds of the genus *Sporophila*: advances in species identification

**DOI:** 10.1007/s11033-026-12530-2

**Published:** 2026-08-06

**Authors:** Júllia Costa dos Reis, Amanda Alves de Melo-Ximenes, Rhewter Nunes, Luiz Alfredo Martins Baptista, Leo Caetano Fernandes Silva, Mariana Pires de Campos Telles

**Affiliations:** 1 Laboratório de Genética e Biodiversidade (LGBio), Instituto de Ciências Biológicas, Universidade Federal de Goiás, Goiânia, Goiás Brasil; 2 Laboratório de Bioinformática e Biodiversidade (LBB), Instituto Acadêmico de Ciências da Saúde e Biológicas, Universidade Estadual de Goiás, UnU de Iporá, Campus Oeste, Iporá, Goiás Brasil; 3 Instituto Brasileiro do Meio Ambiente e dos Recursos Naturais Renováveis (Ibama), Goiânia, Goiás Brasil; 4 Escola de Ciências Médicas e da Vida, Pontifícia Universidade Católica de Goiás, Av. Universitária, 1440, Setor Leste Universitário, Goiânia, Goiás Brasil

**Keywords:** Birds, DNA barcode, Molecular identification, Molecular markers, Primers

## Abstract

**Background:**

Accurate identification of *Sporophila* is crucial for conservation and combating trafficking. Due to morphological similarity among females, molecular tools are essential. Although *cytochrome c oxidase subunit 1* (*COI*) is the standard animal barcode, its limited resolution in recently diverged species necessitates more informative regions, such as *cytochrome b* (*CYTB*) and *NADH dehydrogenase 2* (*ND2*), which are effective for Thraupidae. The aim of this study was to provide molecular mitochondrial markers to support species identification and development of tools that contribute to conservation of *Sporophila*.

**Methods and results:**

The markers were validated using 62 adult males (13 species) and applied to 47 unidentified females from the Centro de Triagem de Animais Silvestres (CETAS). DNA was amplified via PCR, targeting *CYTB* and *ND2*, using genus-specific primers for all samples. *COI* was amplified exclusively for males, using primers based on literature. Sequences were analyzed using an ABI3500.Maximum likelihood trees and haplotype networks compared each studied region. Nearest-neighbor and barcode gap analyses confirmed marker efficiency for species identification, with *CYTB* and *COI* demonstrating the highest reliability. Among the 13 species evaluated, five showed barcode gaps across all markers, while two depended on the marker used. Female samples achieved a 100% amplification rate, with 74.47% showing high identity (> 98%); conclusively, 52.13% were identified with high confidence, whereas the remainder belonged to species falling outside our markers’ current discrimination efficiency.

**Conclusions:**

This study provides efficient tools for future research and conservation initiatives targeting *Sporophila* species.

**Supplementary Information:**

The online version contains supplementary material available at 10.1007/s11033-026-12530-2.

## Introduction

Rapid and accurate species identification is crucial for recognition, management, and conservation. It may also help prevent overexploitation and the hunting of protected species [1, 2]. Thousands of animals are removed from their native ecosystems and confined in captivity [3]. Throughout this process, however, animal specimens are confiscated by enforcement authorities, retrieved, or voluntarily submitted to wildlife rescue and rehabilitation centers. In Brazil, these facilities are known as Centros de Triagem de Animais Silvestres (CETAS) [4], though similar centers operate under different names in other regions of the country, which face comparable challenges wildlife trafficking. CETAS facilities are responsible for receiving, identifying, tagging, sorting, assessing, recovering, rehabilitating, and relocating wildlife specimens [4]. The genus *Sporophila* (family Thraupidae) is among the most frequently seized, due to the attractiveness of its plumage and vocalizations [5]. From 2006 to 2019, CETAS in the state of Goiás (CETAS-GO) received 4,912 seized *Sporophila* individuals, including 22 species and unidentified individuals. The most frequently seized species were *S. angolensis* (2,597 individuals), *S. nigricollis* (748 individuals), and *S. maximiliani* (360 individuals).

The genus *Sporophila* is of commercial value and interest to breeders due to its striking plumage, ease of handling in captivity, and melodious vocalizations [6]. Males are particularly notable for their songs, which are often showcased in competitive singing events and tournaments [7, 8]. According to the International Union for Conservation of Nature (IUCN) criteria, the *Sporophila maximiliani* is endangered [9], with fewer than 100 individuals remaining in the wild in Brazil, while licensed breeding centers house 178,498 specimens, with an even higher number kept in illegal facilities [10]. Due to the high intake rates of *Sporophila* individuals at CETAS facilities and the morphological similarity among females, identifying the species is challenging. Only males display colorful plumage; females exhibit dull, brownish coloration [11]. Given the significant number of specimens received, accurate identification is crucial to ensure proper management and appropriate relocating of the animals.

In these cases, DNA barcoding is an effective method for identifying species, and the mitochondrial gene *cytochrome c oxidase subunit I* (*COI*) is the established barcode marker for the animal kingdom [12]. However, DNA barcoding studies within the genus *Sporophila* have been limited to the southern capuchino seedeater subgroup [13–15], revealing low interspecific differentiation for the *COI* gene. This pattern is attributed to recent divergence, likely within the last 500,000 years, as well as to hybridization in their natural environment [16]. The effectiveness of this specific marker in other species of the genus remains unknown; thus, this study aims to expand the analysis to a broader set.

Studies have shown that improving species identification requires moving beyond a reliance on *COI* gene sequences alone [13]. Additionally, primers commonly referred to as “universal” may not provide sufficient taxonomic coverage or precision [17, 18]. Therefore, it is essential to incorporate additional genetic markers beyond *COI* and use species-specific primers to ensure more robust and accurate identification tailored to the group under study. In this regard, both the mitochondrial genes *cytochrome B* (*CYTB*) and the *NADH dehydrogenase 2* (*ND2*) gene have been successfully used in previous studies to investigate evolutionary relationships and species identification within the Thraupidae family [19–21], being mitochondrial regions with potential for the development of efficient molecular markers for discriminating species within this group.

A highly discriminatory marker should yield a high percentage of intraspecific nearest neighbors; that is, the most similar sequence should belong to an organism of the same species [22]. Furthermore, it should reveal a gap in the barcode gap analysis, indicating a distinct interval (“gap”) between intraspecific and interspecific variation [23, 24]. This interval ensures that intraspecific genetic distances are smaller than interspecific genetic distances. The greater the difference between intraspecific and interspecific genetic variation, the more accurate and successful of molecular makers is in species identification.

Given the impact of wildlife trafficking on the genus *Sporophila* and the scarcity of comprehensive studies employing molecular markers for species identification, this technique is essential for supplementing visual identification and improving accuracy. The present study aims to develop molecular markers to assist in the genetic identification of *Sporophila* species. This study evaluates the performance of three mitochondrial markers (*CYTB*, *COI*, and *ND2*) for species discrimination within the genus. After validating the primers with males identified by morphology, the markers will be used to distinguish females seized and sent to CETAS-GO. These female samples will be compared with sequences available in public database to confirm species identification. This will support accurate species identification and ensure the proper relocating of *Sporophila* individuals.

## Materials and methods

### Sample collection, DNA extraction, PCR amplification, and sequencing

The study was registered in the Sistema Nacional de Gestão do Patrimônio Genético e do Conhecimento Tradicional Associado (SisGen) (ABB582A), and approved by the Comissão de Ética no Uso de Animais (CEUA) of the Universidade Federal de Goiás (UFG) under protocol number 66/23. Blood samples from *Sporophila* individuals were collected in partnership with the CETAS of IBAMA, in the Brazilian states of Goiás (GO) and Rio Grande do Norte (RN), depending on sample availability. A total of 62 adult males representing 13 species were selected based on morphological identification. Up to eight individuals per species were included; when this number was unavailable, all present individuals were sampled. The sampled species and their respective numbers of male individuals were: *S. lineola* (8), *S. maximiliani* (8), *S. nigricollis* (8), *S. albogularis* (7), *S. angolensis* (7), *S. bouvreuil* (6), *S. caerulescens* (6), *S. americana* (3), *S. plumbea* (3), *S. collaris* (2), *S. leucoptera* (2), *S. frontalis* (1), and *S. hypoxantha* (1) (Supplementary Table 1). Additionally, 47 samples from females that could not be morphologically identified to the species level were included. All subsequent molecular analyses were conducted at the Laboratório de Genética & Biodiversidade (LGBio) at UFG.

Blood samples were collected by making a small cut on the bird’s nail and stored on FTA filter paper following the avian blood sampling protocol of the UNIGEN^®^ laboratory [25]. DNA extraction was performed according to the protocol for animal tissue samples [26], with adaptations for filter paper. The samples were homogenized and incubated in a water bath for up to 55 min. Finally, the samples were resuspended in 50 µL of TE buffer (pH 8.0) containing RNase (10 mg/µL). DNA quantification was performed by comparing the samples to concentration markers on a 1% agarose gel stained with ethidium bromide (10 mg/mL).

Species-specific primers were developed for *Sporophila*. First, a search was conducted in the NCBI Nucleotide database to retrieve all available complete mitochondrial genomes available for the Thraupidae family as of October 2019 (Supplementary Table 2). The sequences were then aligned using the MAFFT algorithm [27], and nucleotide diversity (π) was estimated using DnaSP [28]. Regions with high diversity were then analysed using the Basic Local Alignment Search Tool (BLAST) against the NCBI database. This analysis identified the *ND2* and *CYTB* genes as optimal targets. Subsequently, all available partial and complete sequences for these two genes from *Sporophila* representatives were retrieved from NCBI (up to February 2020) (Supplementary Table 3). A nucleotide diversity analysis was performed on the aligned sequences of the genus *Sporophila* to identify regions with the ideal balance for primer design, i.e., conserved sites for annealing of each primer and highly informative adjacent regions capable of distinguishing species.

Primer design was carried out using Geneious Prime v. 2020.0.5 [29], following the settings and conditions described by Melo et al. [18]. To achieve an optimal balance between specificity and amplification efficiency, the following criteria were applied during primer design: degeneracy level below 8; a GC (guanine and cytosine) content between 40% and 60%, and a melting temperature (Tm) difference of less than 5 °C between primer pairs. Forward and reverse primers were developed for both selected genes. Finally, to minimize the risk of experimental failure, we validated the designed primers in silico using the openPrimeR package [30]. This computational step was crucial for evaluating taxonomic coverage and ensuring that the primers recognize all target *Sporophila* species. If also evaluated amplification efficiency, thereby selecting only the most robust candidates for subsequent in vitro PCR validation.

For each molecular marker, PCR was performed in 15 µL reactions containing: 5.77 µL of Milli-Q H₂O; 2.25 µL of DNA at 2.5 ng/µL; 1.13 µL of each primer at 1 µM; 1.5 µL of TP (10X) with MgCl₂; 1.5 µL of BSA (25 mg/mL); 1.5 µL of dNTPs (2.5 µM); and 0.23 µL of *Taq* (*Thermus aquaticus*) DNA polymerase (5 U). The PCR conditions consisted of an initial denaturation cycle at 94 °C for three minutes, 35 cycles of denaturation at 94 °C for one minute, annealing at 50 °C for one minute, and extension at 72 °C for one minute. A final extension cycle was performed at 72 °C for 10 min. PCR products were analyzed by comparing the amplified fragments with the verified against a 1 Kb marker (Invitrogen^®^) on a 1.5% agarose gel stained with ethidium bromide.

The PCR products were purified using Exonuclease I / Shrimp Alkaline Phosphatase (EXO-SAP) enzymes at a 1:9 ratio. The purification process occurred under the following conditions: 37 °C for 94 min, 80 °C for 20 min, then cooled to 4 °C. Next, the sequencing reaction was performed using the Big Dye Terminator v.3 kit (Life Technologies^®^). The reaction mixture consisted of: 3.5 µL of Milli-Q H₂O; 2 µL of Big Dye Buffer; 1 µL of Big Dye Premix; 2.5 µL of primer (forward or reverse) at 1 µM; and 1 µL of purified DNA, for a total volume of 10 µL. The cycling conditions included 25 cycles of 95 °C for 20 s, 50 °C for 15 s, and 60 °C for one minute.

The sequencing reaction was precipitated by adding 1 µL each of EDTA (125 mM, pH 8.0) and sodium acetate (3 M) and 25 µL of 100% ethanol. The mixture was then vortexed for 10 s and incubated at room temperature for 15 min. The samples were then centrifuged at 2,000 RCF for 45 min at 4 °C. After removing the seal, the plate was inverted and spun at 700–800 RPM. Then, 30 µL of 70% ethanol was added, followed by to vortex for 10 s and centrifugation under the same conditions for 15 min. After inverting the plate and performing another inverted spin, the samples were dried in a thermal cycler at 60 °C for 10 min with the lid open. The samples were eluted in 10 µL of Hi-Di formamide, vortexed for 10 s, and then denatured at 95 °C for three minutes. Capillary electrophoresis was performed using an ABI 3500 automated genetic analyzer (Applied Biosystems^®^). The consensus sequence was obtained from two sequencing reactions, forward and reverse.

We analyzed the sequencing results using the Sequencing Analysis software (Applied Biosystems^®^) to assess sequence quality. Base calling and consensus sequence determination were performed using SeqScape v3.0 (Applied Biosystems^®^). Polymorphisms were identified by comparing the analyzed sequences to the reference sequence. The reference sequences used were for the *CYTB* gene (GU215316.1), the *ND2* gene (FJ176155.1), and the *COI* gene (MK433296.1) of *Sporophila angolensis*, retrieved from GenBank.

### Data analysis

Multiple sequence alignment was conducted using ClustalW in BioEdit 7.2.5 [31]. Manual editing to remove primer and standardize lengths. The *CYTB*, *ND2*, and *COI* gene sequences were then concatenated using Sequence Matrix 1.9 v1.7.6 software [32].

We reconstructed the phylogeny of *Sporophila* for each gene using a maximum likelihood tree. To be more representative, considering the number of species sequenced in this study, we used data available in the NCBI nucleotide database (Supplementary Table 4). For each species, whenever possible, we selected up to 8 sequences to be more representative. Without considering the outgroup, a total of 182 *Sporophila* sequences were used for the *CYTB* gene (62 sequenced + 120 sequences from the database) representing 35 species, 134 for the *ND2* gene (62 sequenced + 72 sequences from the database) representing 33 species, and 220 sequences for the *COI* gene (62 sequenced + 158 sequences from the database) representing 29 species. The identifiers of the sequences used for each gene are in the Supplementary Table 4. Maximum likelihood tree construction was performed using IQ-TREE v. 2.2.0 [33], using ModelFinder Plus [34] to determine the most suitable nucleotide model (*CYTB* gene: HKY + F+G4; *ND2* gene: TN + F+G4; *COI* gene: TPM2 + F+G4) and 1,000 bootstrap replicates. In addition, for the 19 species containing sequences of the three genes, analyses of the concatenated genes were considered, with the ModelFinder Plus analysis considering the gene partitions (*CYTB* gene: HKY + F+G4; *ND2* gene: K3Pu + F+G4; *COI* gene: TPM2u + F+I+G4) and 1,000 bootstrap replicates. The outgroup was *Saltator similis* (Passeriformes: Thraupidae), and the sequences were retrieved from GenBank (gene *CYTB*: MK419316.1; gene *ND2*: MK419316.1; gene *COI*: MK419316.1). The resulting phylogenetic tree was plotted using iTOL v. 6 [35].

In order to verify and compare the species considering each region of the molecular markers, and the concatenated sequences. A haplotype network was constructed for each analyzed gene with Median-Joining and for the concatenated sequences, considering the samples that we sequenced in the present study, using Network v.10.2.0.0 software [36]. We used genetic distance, calculated from the concatenated sequences, to assess correct specimen identification and compare boundaries among specimens. The Kimura 2-parameter (K2P) model [37] was employed in the distance-based approach because it is considered the most suitable metric for low genetic distances [38]. Sequence divergence among individual concatenated sequences was calculated in R using the “ape” package and the “dist.dna” function [39, 40]. In addition, we performed paired distance matrix analysis for each individual gene region.

We evaluated the efficiency of the molecular markers both individually and using concatenated sequences by comparing intraspecific identification through the nearest neighbor test and the Barcoding Gap analysis. In the nearest neighbor test, each sequence is compared to all the others to identify the smallest genetic distance. However, species represented by only one sequence (*Sporophila frontalis* and *Sporophila hypoxantha*) may yield unreliable results. We performed the nearest neighbor test using the “nearNeighbor” function in R, with the “Spider” package, to determine if sequences with the lowest divergence belonged to the same species [41]. The identification success rate was calculated by dividing the number of nearest neighbor matches by the total number of sequences. The barcoding gap was assessed by comparing the minimum interspecific divergence and maximum intraspecific divergence. These were calculated using the “nonConDist” and “maxInDist” functions from the “Spider” package in R [41]. The K2P model was also applied. Additionally, a barcoding gap histogram was generated using the “barcoding.gap” function from the “BarcodingR” package, employing the K2P model [42].

Species identification was performed by comparing the *CYTB*, *ND2*, and *COI* gene sequences from males to reference sequences from the GenBank using the Basic Local Alignment Search Tool (BLAST). The *COI* sequences were also referenced in the Barcode of Life Data System (BOLD). Percent identity was considered, and matches above 98.00% with reference sequences from the same species were accepted. Female sequences were compared to reference sequences via BLAST using the same ≥ 98.00% percent identity criterion. The results were organized in spreadsheets and analyzed in R to generate graphs that facilitated data visualization [39].

## Results

Based on both in silico analyses and experimental sequencing, primer combinations were selected for their optimal performance the ability to amplify the largest region of each gene (Supplementary Table 5). For the *CYTB* gene, the CYT-A primer set was chosen. This set includes the forward primer SpoCTYB-01 F (5’-CCCTCATAGCAACAGCCTT-3’, 19 bp) and the reverse primer SpoCTYB-03R (5’-GAATGGGTGTTCTACGGGTT-3’, 20 bp). The expected PCR product size is 676 bp. The selected primer set for the *ND2* gene was ND-D, including the forward primer SpoND2-02 F (5’-GAGCCATTGAAGCTGCCACT-3’, 20 bp) and the reverse primer SpoND2-02R (5’-GCTGTTGGGGCTATATCTTG-3’, 20 bp). This set generates a 737 bp amplicon.

A total of 62 DNA samples were extracted and amplified from adult male birds representing 13 species of the *Sporophila* genus. The species-specific primers designed for the *CYTB* and *ND2* genes successfully amplified all samples, achieving 100% efficiency. Initial amplification of the *COI* gene was performed using the BirdF1 (5’-TTCTCCAACCACAAAGACATTGGCAC-3’, 26 bp) and BirdR1 (5’-ACGTGGGAGATAATTCCAAATCCTG-3’, 25 bp) primer set, which successfully amplified 49 samples with 79.03% efficiency [43]. The remaining 13 samples (20.97%) were subsequently amplified using the FishF1 (5’-TCAACCAACCACAAAGACATTGGCAC-3’, 26 bp) and FishR1 (5’-TAGACTTCTGGGTGGCCAAAGAATCA-3’, 26 bp) primers [44]. Sequencing yielded amplicons with an average length of 600 bp (range: 513–663 bp) for *CYTB*, 565 bp (range: 496–651 bp) for *ND2*, and 587 bp (range: 505–695 bp) for *COI*. After editing and aligning the sequences, they were trimmed to standardized lengths of 457 bp, 417 bp, and 412 bp for the *CYTB*, *ND2*, and *COI* genes, respectively. The generated sequences have been deposited in the NCBI Nucleotide database and can be accessed under the following accession numbers: PZ321016–PZ321124 (CYTB), PZ321125–PZ321233 (ND2) and PZ295779–PZ295840 (COI).

**Fig. 1:**
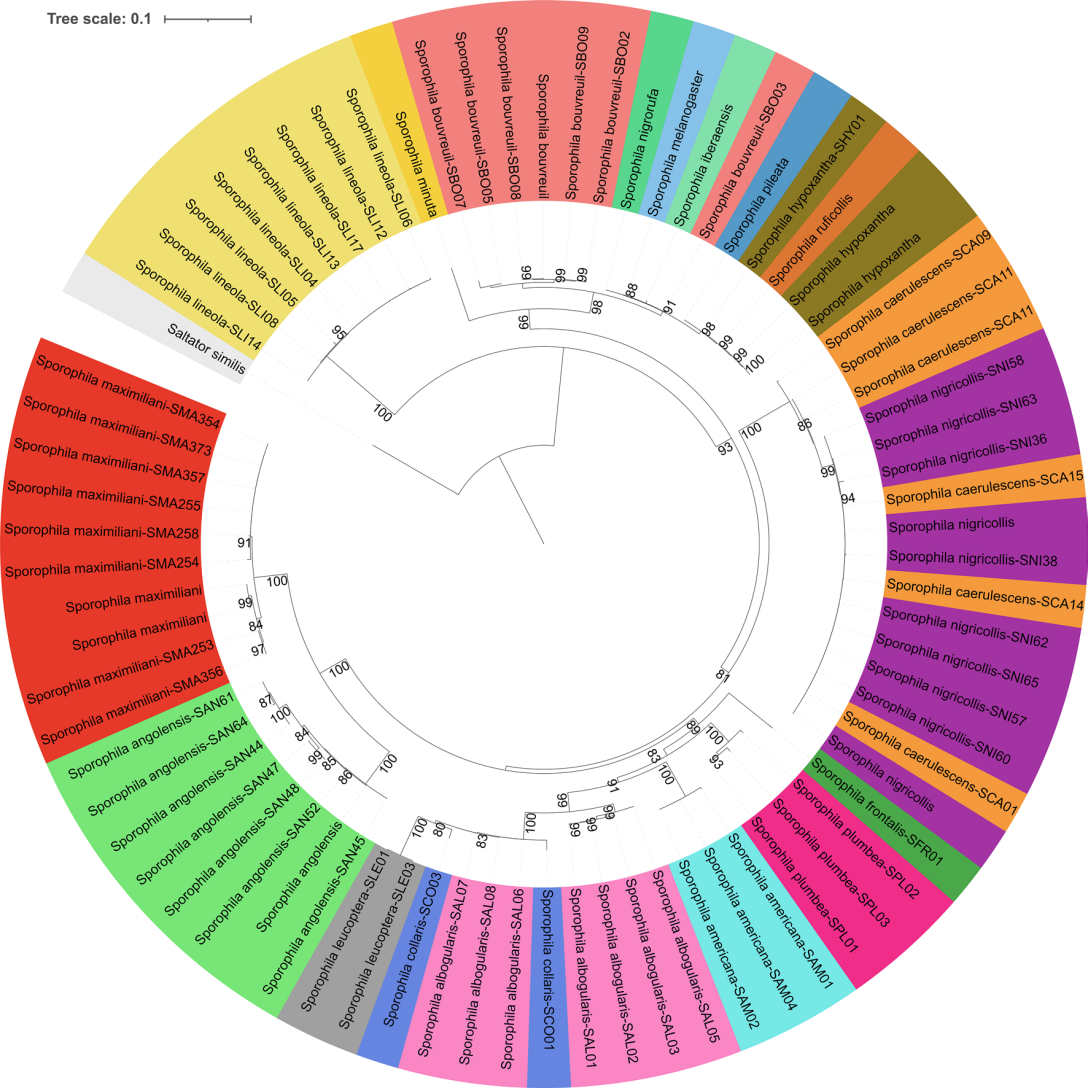
Maximum likelihood tree based on mitochondrial data from three concatenated molecular markers (*CYTB*, *ND2*, and *COI*) from 19 species within the genus *Sporophila*, number of characters used 1328. The numbers on the branches indicate bootstrap support based on 1,000 replicates. The colors correspond to each species

The maximum likelihood tree was performed using the concatenated sequences of individuals sequenced in this study and of sequences available in Nucleotide NCBI (Fig. 1), *CYTB* gene (Supplementary Fig. 1), *ND2* gene (Supplementary Fig. 2), and gene *COI* (Supplementary Fig. 3). It is evident that topological inconsistencies exist between the concatenated tree and the individual results of each molecular marker for some species. Conversely, consistency was observed in the sister-group relationships between *S. angolensis* and *S. maximiliani*, as well as between *S. caerulescens* and *S. nigricollis*. Although many individuals belonging to the same species clustered as expected, some species failed to exhibit clearly defined phylogenetic clades. Species groups such as *Sporophila caerulescens* and *S. nigricollis*, *S. albogularis* and *S. collaris*, and the southern capuchino complex (including *S. bouvreuil* e *S. hypoxantha*) reflected low genetic distances, suggesting a close evolutionary proximity.

**Fig. 2:**
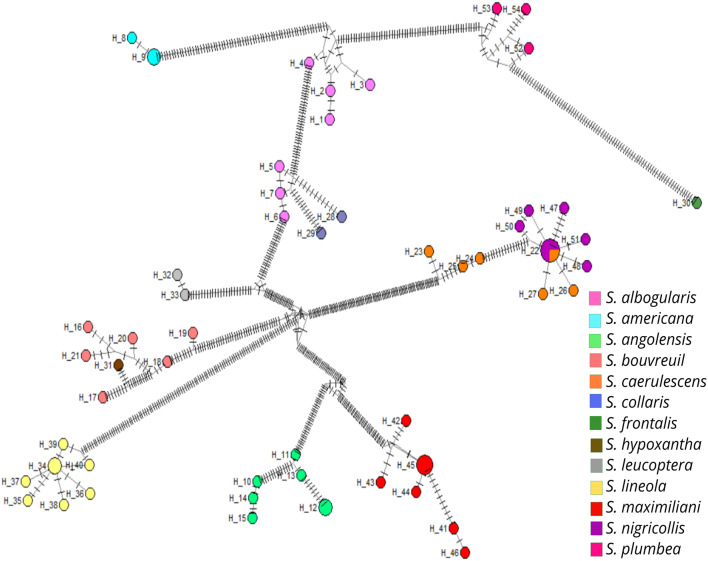
Mitochondrial haplotype network for 62 adult male individuals from 13 species of the genus *Sporophila*, based on the concatenated sequences (*CYTB*, *ND2* and *COI*), number of characters used 1287. Haplotypes are represented by circles, with circle size proportional to the number of individuals carrying a given haplotype, and color indicating species identity according to the legend. Linear marks on the lines connecting haplotypes represent substitutions separating them

A haplotype network visualization was conducted for each mitochondrial region using the analyzed markers. Of the 62 individuals representing 13 species, 33 haplotypes were identified for the *CYTB* gene (Supplementary Fig. 4), 34 for *ND2* (Supplementary Fig. 5), 38 for *COI* (Supplementary Fig. 6), and 54 for the concatenated sequences (Fig. 2). Most individuals of the same species clustered together, with a few substitutions observed among haplotypes. In contrast, individuals from different species generally exhibited numerous substitutions. However, in some cases, individuals from distinct species showed few substitutions, for example, *Sporophila albogularis* and *S. collaris*. Only *S. caerulescens* and *S. nigricollis* shared haplotypes across all markers. Haplotype sharing was also observed between *S. albogularis* and *S. collaris* for *CYTB*, and among *S. albogularis*, *S. collaris*, and *S. leucoptera* for *ND2*.

The mean intraspecific and interspecific genetic distances were calculated from the concatenated sequences using a pairwise comparison matrix based on the average intra- and interspecific distances among individuals of each species (Table 1). In addition, a pairwise distances matrix was generated for each gene included, gene *CYTB* (Supplementary Table 6) gene *ND2* (Supplementary Table 7), and gene *COI* (Supplementary Table 8). The minimum intraspecific distance was 0.0008 for *S. leucoptera*, and the maximum was 0.0220 for *S. albogularis*, giving an overall mean of 0.0085 (0.85%). The minimum interspecific distance was 0.0086 between *S. caerulescens* and *S. nigricollis*, and the maximum was 0.1250 between *S. lineola* and *S. nigricollis*, with an overall mean of 0.0893 (8.93%). Although interspecific distances were generally higher than intraspecific ones, relatively low interspecific values were observed between *S. albogularis* and *S. collaris* (0.0321), *S. hypoxantha* and *S. bouvreuil* (0.0169), and *S. nigricollis* and *S. caerulescens* (0.0086). Intraspecific distances could not be calculated for *S. frontalis* and *S. hypoxantha*, as only one individual from each species was analyzed.

**Table 1:** Pairwise distances matrix showing the average intraspecific and interspecific genetic distances among the 13 *Sporophila* species, values in bold along the diagonal represent mean intraspecific genetic distances, while off-diagonal values represent interspecific genetic distances, calculated using the K2P model from the concatenated sequences. The number of characters used is 1287

Species	1	2	3	4	5	6	7	8	9	10	11	12	13
1. *S.albogularis*	**0.0220**												
*2. S.americana*	0.0626	**0.0010**											
*3. S.angolensis*	0.0918	0.0977	**0.0097**										
*4. S.bouvreuil*	0.0830	0.0877	0.0989	**0.0119**									
*5. S.caerulescens*	0.0900	0.0935	0.0983	0.0902	**0.0101**								
*6. S.collaris*	0.0321	0.0721	0.0957	0.0884	0.0956	**0.0190**							
*7. S.frontalis*	0.0817	0.0829	0.0932	0.0900	0.0912	0.0950	**NA**						
*8. S.hypoxantha*	0.0816	0.0853	0.1004	0.0169	0.0915	0.0861	0.0938	**NA**					
*9. S.leucoptera*	0.0641	0.0895	0.0959	0.0798	0.0964	0.0480	0.0976	0.0806	**0.0008**				
*10. S.lineola*	0.1172	0.1192	0.1199	0.1020	0.1244	0.1206	0.1087	0.1031	0.1163	**0.0023**			
*11. S.maximiliani*	0.0905	0.0919	0.0712	0.0926	0.0984	0.0945	0.0945	0.0967	0.0903	0.1130	**0.0055**		
*12. S.nigricollis*	0.0911	0.0958	0.0998	0.0926	0.0086	0.0952	0.0942	0.0937	0.0961	0.1250	0.1016	**0.0025**	
*13. S.plumbea*	0.0606	0.0651	0.0920	0.0841	0.0888	0.0698	0.0714	0.0814	0.0847	0.1082	0.0889	0.0902	**0.0084**

For S. frontalis and S. hypoxantha, the intraspecific distance was considered not applicable (NA) as the analysis was based on a single individual

Analysis of the efficiency of molecular markers showed that, based on the nearest neighbor approach for the *CYTB*, *COI*, and concatenated sequences, 87.10% (54 sequences) exhibited lower genetic distances with individuals of the same species. Examples of genetic proximity to individuals of other species included *S. bouvreuil* with *S. hypoxantha*, *S. caerulescens* with *S. nigricollis*, and *S. collaris* with *S. albogularis*. Additionally, *S. hypoxantha* was found to be genetically close to *S. bouvreuil* for all markers. For the *ND2* gene, 82.26% (51 sequences) showed intraspecific proximity, with interspecific neighborhood patterns similar to those previously observed. These patterns additionally included *S. leucoptera* with *S. albogularis*.

**Fig. 3:**
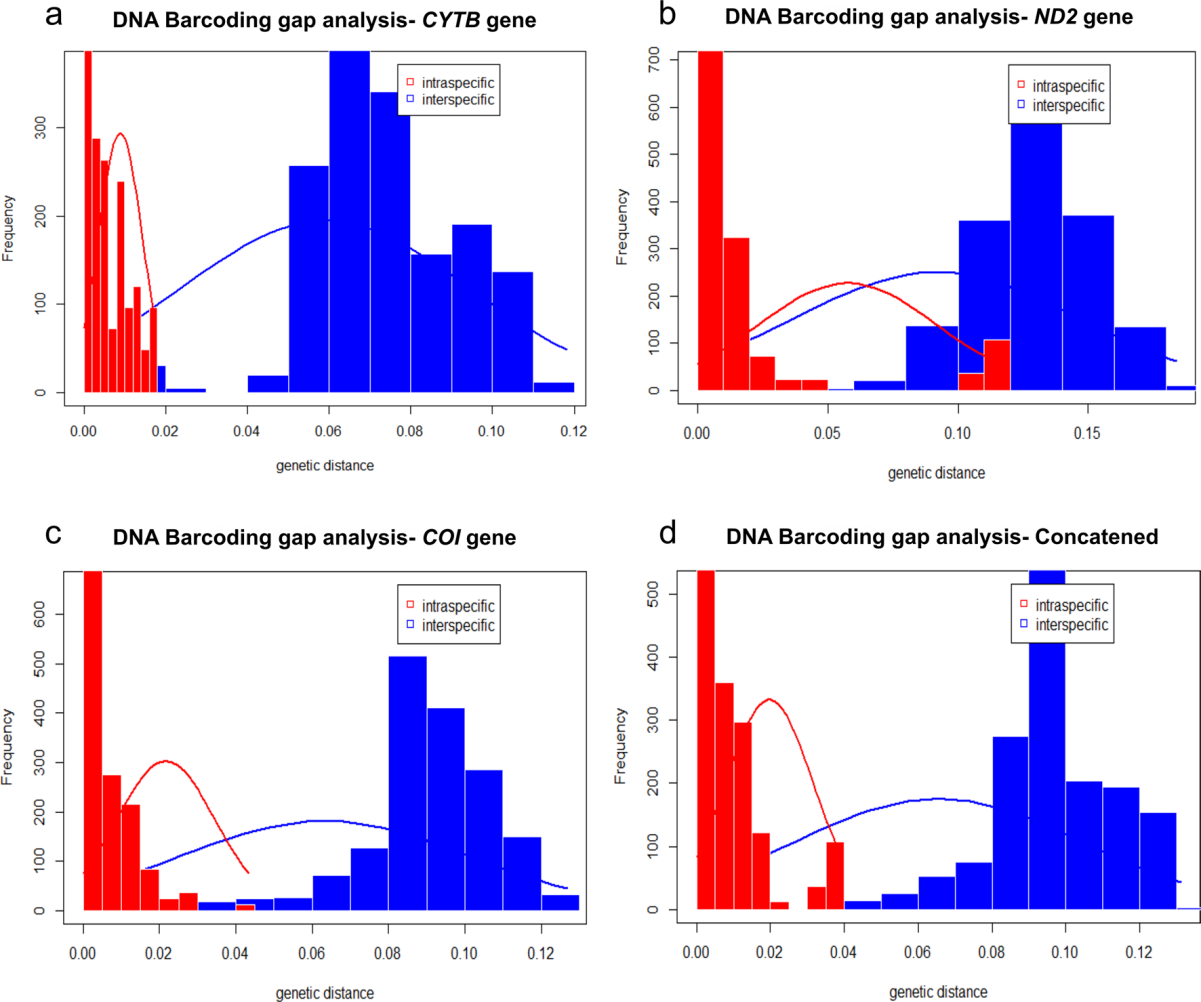
Barcoding Gap including the 13 *Sporophila* species, with sequence lengths of 457 bp, 417 bp, and 412 bp for the *CYTB*, *ND2*, and *COI* genes, respectively, yielding a total of 1,287 characters in the concatenated alignment. Histograms showing intraspecific distances in red and interspecific distances in blue for the regions analyzed. In all cases, despite some overlap, the variation in interspecific distance was considerably greater

Barcoding Gap analysis involving 13 species (Fig. 3) revealed that, while there was some overlap between intra- and interspecific distances for all markers, the latter showed greater variation and higher averages. For *CYTB*, intraspecific distances ranged from 0% to 1.7% (mean 0.4%), whereas interspecific distances ranged from 0% to 11.8% (mean 7.2%). For *ND2*, intraspecific distances ranged from 0% to 11.5% (mean 1.4%), while interspecific distances ranged from 0% to 18.3% (mean 12.4%). For *COI*, intraspecific distances ranged from 0% to 4.3% (mean 0.5%), and interspecific distances from 0% to 12.6% (mean 8.8%). In concatenated sequences, while intraspecific distances ranged from 0% to 3.9% (mean 0.8%), and interspecific distances ranged from 0% to 13.1% (mean 9.3%).

**Fig. 4:**
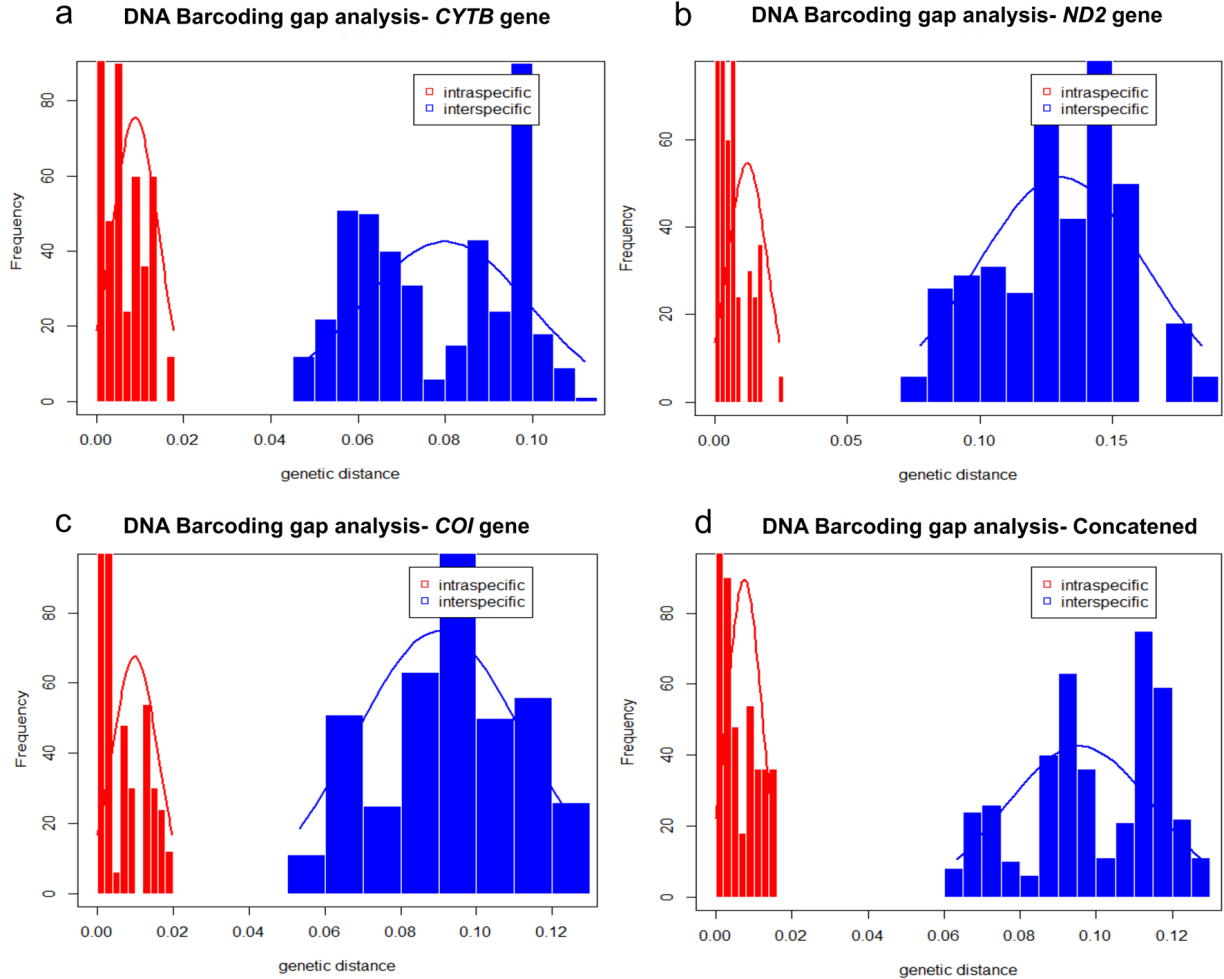
Barcoding Gap histograms with *S.americana*, *S. angolensis*, *S. frontalis*, *S. leucoptera*, *S.lineola*, *S. maximiliani* e *S. plumbea*. Sequence lengths of 457 bp, 417 bp, and 412 bp for the *CYTB*, *ND2*, and *COI* genes, respectively, yielding a total of 1,287 characters in the concatenated alignment. Showing intraspecific distances in red and interspecific distances in blue. When excluding the six species that exhibited the lowest interspecific distances, a clear gap between intraspecific and interspecific distances was observed across all markers

Excluding six species with low interspecific distances and haplotype overlap (*Sporophila albogularis*, *S. collaris*, *S. hypoxantha*, *S. bouvreuil*, *S. nigricollis*, and *S. caerulescens*), the barcoding gap analysis with the remaining seven species (*S. americana*, *S. angolensis*, *S. frontalis*, *S. leucoptera*, *S. lineola*, *S. maximiliani*, and *S. plumbea*) (Fig. 4) revealed a clear separation between intra- and interspecific distances across all markers. For *CYTB*, intraspecific distances ranged from 0% to 1.7% (mean 0.5%), while interspecific distances ranged from 4.7% to 11.2% (mean 7.5%). For *ND2*, intraspecific distances ranged from 0% to 2.4% (mean 0.5%), while interspecific distances ranged from 7.7% to 18.3% (mean 13.0%). For *COI*, the range of intraspecific distances was from 0% to 1.9% (mean 0.5%), and the range of interspecific distances was from 5.3% to 12.6% (mean 9.3%). In concatenated sequences, intraspecific distances ranged from 0% to 1.4% (mean 0.5%), while interspecific distances ranged from 6.3% to 12.7% (mean 9.9%).

**Fig. 5:**
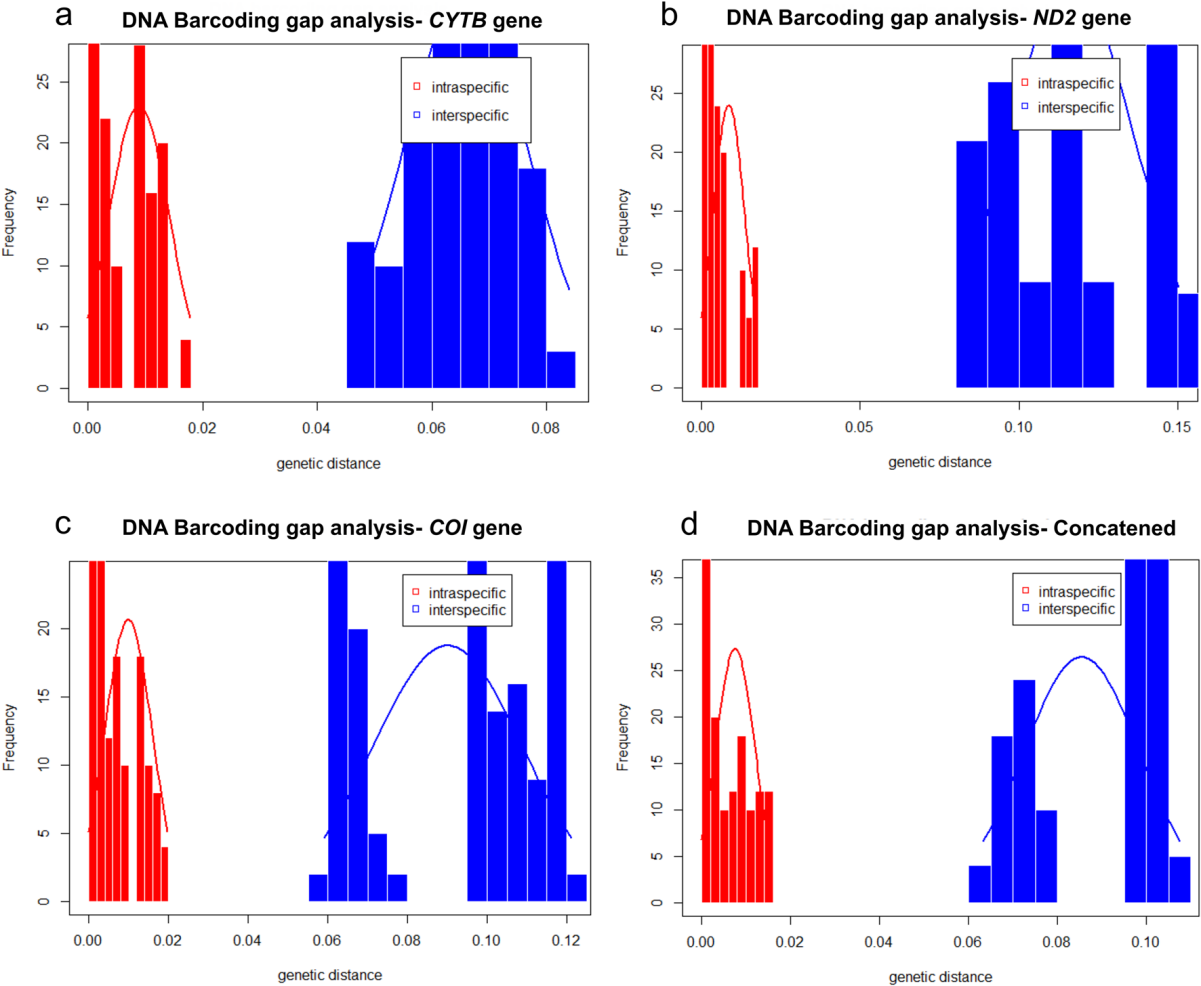
Barcoding Gap most frequently seized species at CETAS-GO, *S. angolensis*, *S. maximiliani*, *S. nigricollis*. Sequence lengths of 457 bp, 417 bp, and 412 bp for the *CYTB*, *ND2*, and *COI* genes, respectively, yielding a total of 1,287 characters in the concatenated alignment. Histograms considering only the three most frequently seized species in the state of Goiás, for each marker analyzed and for the concatenated sequences. The histograms show intraspecific distances in red and interspecific distances in blue for the regions studied, as well as the gap between these distances across all markers

When calculating the barcoding gap for the three most frequently seized species in the state of Goiás (*Sporophila angolensis*, *S. maximiliani*, and *S. nigricollis*) (Fig. 5), clear separation was observed between intra- and interspecific distances across all markers, indicating high discrimination efficiency. For *CYTB*, intraspecific distances reached 1.7% (mean 0.5%), while interspecific distances ranged from 4.7% to 8.3% (mean 6.5%). For *ND2*, intraspecific distances reached up to 1.7% (mean 0.4%), while interspecific distances ranged from 8.2% to 15% (mean 12%). For *COI*, intraspecific distances reached up to 1.9% (mean 0.6%), while interspecific distances ranged from 5.8% to 12.1% (mean 9.3%). In concatenated sequences, intraspecific distances reached 1.4%, while interspecific distances ranged from 6.3% to 10.7% (mean 9.1%). Thus, it is evident that, for the most frequently seized species at CETAS-GO, all markers demonstrate a gap between intraspecific and interspecific distances, effectively discriminating between species within the genus *Sporophila*.

Calculating the difference between intraspecific and interspecific genetic distances allowed us to determine the barcoding gap value for each of the 11 *Sporophila* species (excluding *S. frontalis* and *S. hypoxantha* due to a lack of intraspecific distance data). This analysis helps identify species with clear genetic differentiation, facilitating their recognition and distinction (Table 2). The barcoding gap was detected in 60% of *CYTB* sequences, 48.33% of *ND2* sequences, 63.33% of *COI* sequences, and 58.33% of concatenated sequences. This indicates that a single marker may be sufficient to distinguish genetically distinct species. Of the 13 evaluated species, five (*S. americana*, *S. angolensis*, *S. lineola*, *S. maximiliani*, and *S. plumbea*) exhibited a barcoding gap across all markers. *S. caerulescens*, *S. collaris*, and *S. nigricollis* showed no gap in any marker. *S. albogularis* and *S. bouvreuil* presented partial results depending on the marker.

**Table 2:** The 13 species used in the study with each gene corresponding to a marker. The symbols in the table mean:✓ all individuals of the species showed a barcoding gap; X no individuals of the species showed a barcoding gap; ? some individuals of the species showed a barcoding gap, while others did not

Species	Species with evidence of a barcoding gap			
	CYTB	ND2	COI	Concatenated
*1. S.albogularis*	X	X	✓	?
*2. S.americana*	✓	✓	✓	✓
*3. S.angolensis*	✓	✓	✓	✓
*4. S.bouvreuil*	?	X	X	X
*5. S.caerulescens*	X	X	X	X
*6. S.collaris*	X	X	X	X
*7. S.frontalis*	NA	NA	NA	NA
*8. S.hypoxantha*	NA	NA	NA	NA
*9. S.leucoptera*	✓	X	✓	✓
*10. S.lineola*	✓	✓	✓	✓
*11. S.maximiliani*	✓	✓	✓	✓
*12. S.nigricollis*	X	X	X	X
*13. S.plumbea*	✓	✓	✓	✓

*Sporophila albogularis* showed no barcoding gap in any sequences for the *CYTB* and *ND2* gene markers. Detailing the cases in which only some sequences from certain species exhibited a barcoding gap. In contrast, all *COI* gene sequences and some concatenated sequences (four out of seven individuals) exhibited a gap. For *S. bouvreuil*, the barcoding gap was observed only in the *CYTB* gene, demonstrating its effectiveness for most individuals (five out of six). Additionally, *S. leucoptera* exhibited a gap across all markers except *ND2*.

Comparing the obtained sequences with the GenBank and BOLD databases revealed that BOLD (Supplementary Table 9) has no data for the *CYTB* and *ND2* genes of the *Sporophila* genus. This is consistent with the BOLD consortium’s standard, which recognizes only *COI* gene sequences as the official DNA barcode for vertebrates. Overall, 82.80% (154 sequences out of a total of 186) of the 62 adult male samples were identified with an identity percentage equal to or above 98.00%, of which 148 sequences corresponded to the previously made morphological identification. Of the sequences with an identity percentage lower than 98.00%, we obtained a total of 16.13% of sequences. The lack of percentage identity with our sample may be attributed to non-overlapping target regions within the gene, high levels of intraspecific phylogeographic divergence between populations, possible sequencing /identification errors in the public record, or a lack of data in the databases. For example, when searching for *S. frontalis*, we found only one 210 bp sequence related to the *CYTB* gene, while when searching for *S. albogularis*, we found no deposited sequences for the *COI* gene, just as there are no sequences for the *CYTB* region of the species *S. americana*.

**Fig. 6:**
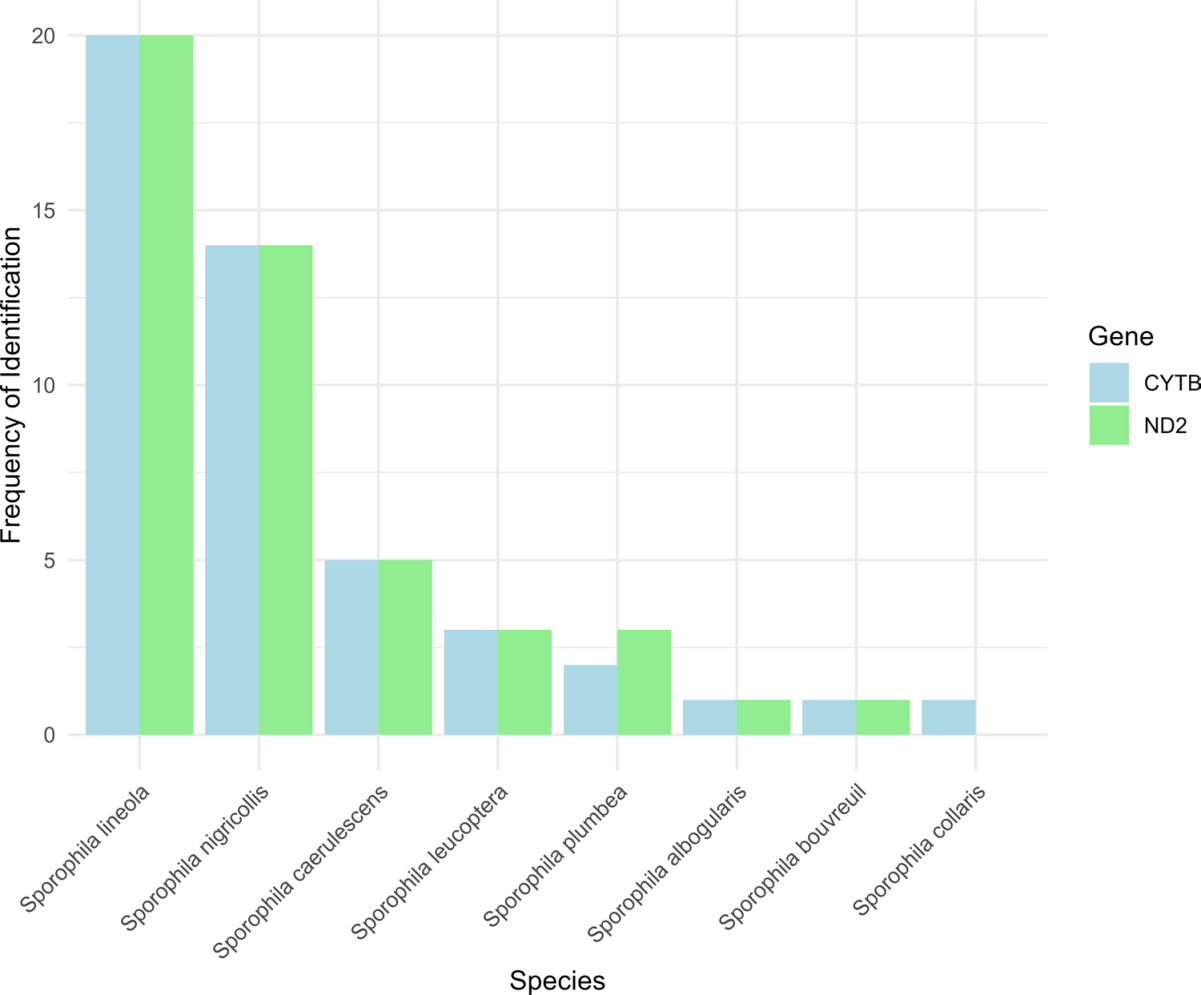
Identification frequency of different species within the genus *Sporophila* using the mitochondrial genes *CYTB* (blue) and *ND2* (green). The bars show the number of samples identified for each species and highlight the efficiency of each gene in discriminating between species. Depending on the marker used, *S. collaris* and *S. plumbea* showed similarities with different species. The number of characters is indicated for each sequence in Supplementary Table 10

Of the 47 female samples lacking prior identification, DNA extraction and amplification were successfully achieved for both *CYTB* and *ND2* genes (100%), resulting in sequences ranging from 503 to 637 bp and 446–659 bp, respectively. BLAST analysis (Supplementary Table 10) revealed that 20 samples showed percent identity with *S. lineola*, 14 with *S. nigricollis*, five with *S. caerulescens*, three with *S. leucoptera*, two with *S.plumbea*, one with *S. bouvreuil* and one with *S. albogularis* (Fig. 6). One individual was identified as belonging to different species by the markers. SPO17 showed greater percent identity with *S. collaris* (95.29%) for the *CYTB* gene and with *S. plumbea* (92.63%) for the *ND2* gene. Overall, 74.47% of the 94 obtained sequences showed percent identity equal to or greater than 98%. However, 25.53% falling below 98% (3 in *CYTB* and 21 in *ND2*).

## Discussion

A total of 62 adult male *Sporophila* individuals from 13 species were sequenced using regions of the *CYTB*, *ND2*, and *COI* genes to evaluate their potential as effective molecular markers for species identification. Sample collection for *Sporophila* is limited because blood is rarely obtained and most confiscated birds are quickly released by CETAS, which makes scientific studies challenging. DNA barcoding methodology requires that DNA fragments be easily amplified to ensure applicability [45]. The genus specific primers designed for the *CYTB* and *ND2* genes demonstrated high amplification efficiency. In contrast, the *COI* region showed partial efficiency with the BirdF1 and BirdR1 primers. For the remaining samples, the FishF1 and FishR1 primers were necessary. These results highlight the limitations of “universal” primers and reinforce the idea that their coverage may be narrower than initially anticipated [18]. Given the superior amplification performance of the genus-specific primers, we propose that *CYTB* and *ND2* serve as effective supplementary DNA barcodes for *Sporophila*.

The maximum likelihood tree while most specimens of the same species were grouped together, some clades were not well defined. These include *S. caerulescens* and *S. nigricollis*, *S. albogularis* and *S. collaris*, as well as, southern capuchino seedeaters, including *S. bouvreuil* and *S. hypoxantha*. These groupings highlight the genetic similarity among closely related species and are consistent with phylogenetic studies suggesting close relationships between them [16, 46]. The haplotype network, shows that *S. caerulescens* and *S. nigricollis* are the only species that share haplotypes across all markers, this sharing occurs because both species are very closely related, having separated recently in evolution, and can interbreed in nature. As a practical impact on the relocating of individuals, this extreme similarity prevents molecular markers from differentiating the two species. This results in a diagnostic conflict, making it impossible to correctly assign or relocating a specimen to its respective taxonomic species when using only these genes in isolation.

The paired distance matrix of the concatenated sequences revealed that intraspecific distances ranged from 0.08% to 3.94% (mean = 0.85%), while interspecific distances ranged from 0.00% to 13.14% (mean = 8.93%). These values extend the divergence limits previously reported by Campagna et al. (2010), who used the *COI* marker in capuchino (*S. castaneiventris* and *S. minuta*) for the genus *Sporophila* (intra: 0.12%–0.37%; inter: 6.5%–9.3%) [14]. This greater range of variation and, notably, the absence of minimal interspecific divergence (0.00%) in our data reflect the evolutionary complexity of the group. The overlap between intra- and interspecific distances reveals a narrow or non-existent barcode gap in certain taxa, a characteristic pattern of lineages that have undergone recent evolutionary radiation and hybridization, where ancestral polymorphisms are still shared. Given this heterogeneity and points of overlap, it becomes essential to investigate individually which species maintain the barcode gap and which fail in taxonomic discrimination.

This study analyzed the species *S. hypoxantha* belonging to the southern capuchino seedeaters, and *S. bouvreuil*, considered a sister species to the southern capuchino seedeaters [15, 47]. The low interspecific distance (0.017) between these species indicates limited genetic variation, which is possibly due to a recent common ancestry and hybridization events [16]. While *S. bouvreuil* is no longer classified within the southern seedeaters, being currently positioned as a sister lineage to this clade, both species still retain a remarkably high level of genetic similarity. However, only one *S. hypoxantha* individual was analyzed, which may limit the results. Additionally, we observed low genetic distances between *S. albogularis* and *S. collaris* (0.032), and between *S. nigricollis* and *S. caerulescens* (0.009).

Most species exhibited a high rate of intraspecific nearest neighbors, indicating that the genetic markers (*CYTB*, *COI*, and *ND2*) effectively differentiate the majority of *Sporophila* species. Where intraspecific neighbors were absent, low interspecific distances were observed, suggesting limited genetic variation between those species. This reinforces the idea that despite the markers’ overall effectiveness, recent evolutionary divergence and genetic proximity still pose challenges to distinguishing certain species [14, 16].

In the barcoding gap analysis of the 13 *Sporophila* species, there was overlap in distances for all markers. This overlap is due to the species’ recent divergence and genetic proximity, which limited the time for the significant differences to accumulate [16]. Excluding species with low interspecific distance and haplotype sharing revealed a clear barcode gap for *S. americana*, *S. angolensis*, *S. frontalis*, *S. leucoptera*, *S. lineola*, *S. maximiliani*, and *S. plumbea* across all markers. The results regarding the most frequently seized species at CETAS-GO demonstrate the efficiency of these markers in discriminating species. Thus, the molecular markers described in this study are particularly valuable for identifying most of the species that arrive at CETAS through seizures and should be applied to other species of the genus that also have high seizure rates and are targeted in other regions. When applied, these markers will directly impact the proper enforcement of fines, the appropriate management of the animals, and their correct relocating. Given the high success rate of discriminating seizes specimens, we reinforce the role of the *CYTB* and *ND2* genes as effective DNA barcodes for this genus.

The inability of a specific marker to distinguish between species is due to biological processes inherent to the group being studied. Recent studies on mitochondrial genomes of *Sporophila* species report that mitogenomes exhibit a highly conserved pattern, maintaining gene structure and order, while also showing low nucleotide diversity. Consequently, they suggest that complete mitogenome sequencing can be applied to improve, compare, and molecularly differentiate these species [48] However, this approach may not apply to taxa lacking a clear barcoding gap. For instance, *S. iberaensis* and *S. hypoxantha* exhibit extremely low genomic differentiation and no mitochondrial divergence, indicating that increasing the number of analyzed characters does not resolve the lack of genetic resolution [49]. This genetic similarity and lack of resolution are primarily driven by the species’ recent evolutionary origin and ongoing hybridization events, which prevent significant mutation accumulation [16]. Although whole-genome studies on southern capuchinos make it possible to identify discriminatory regions and develop targeted primers for species differentiation, this strategy remains less practical and economically viable for routine use [50]. Therefore, identifying specific genomic regions capable of successfully discriminating species is a challenge and becomes essential, which led us to test these specific molecular markers. Given the complex evolutionary context of this genus, the identification of markers capable of distinguishing at least the species most targeted by illegal wildlife trafficking represents a significant and valuable advance for the application of conservation measures.

The BLAST results confirmed that the *CYTB* and *COI* genes were more effective at identifying adult males than the *ND2* gene. This underscores the need to increase the representation of sequences in the GenBank and BOLD databases. Without robust, comprehensive reference databases, species identification is limited, which compromises biodiversity, conservation, and ecology studies [51]. Therefore, it is crucial to expand and update these databases to ensure their reliability and accuracy in species identification [52]. A lack of reference data, particularly for specific genes, hinders the correct and efficient identification of species, which is crucial for the conservation of the *Sporophila* genus.

The molecular markers *CYTB* and *ND2* were used with specific primers for application in the identification of female specimens of the genus *Sporophila*, which were compared with sequences in the database to assess the percentage of identity. The amplification success rate was 100%, and the identification percentage was equal to or above 98% in 74.47% of the sequences. It is important to consider the specific cases revealed by the BLAST results. For example, the percent identity of SPO17 with *S. collaris* (95.29%) for the *CYTB* gene and with *S. plumbea* (92.63%) for the ND2 gene, are too low for reliable species identification. There are two hypotheses that could explain this result. The first is that the specimen belongs to a species for which there are no reference sequences for these markers in GenBank, resulting in alignment with the most genetically similar available species. The second hypothesis is that the SPO17 specimen belongs to a species within the *Sporophila* genus that cannot be accurately discriminated by the *CYTB* and *ND2* genes. Examples, of such species include *S. albogularis*, *S. bouvreuil*, *S. caerulescens*, *S. collaris*, *S. frontalis*, *S. hypoxantha* and *S. nigricollis*, as well as some species not addressed in the present study.

In addition, the ND2 marker samples showed some sequences with identity percentages below 98%, mainly for the species *S. lineola*. The database was consulted, and we observed that there is only one reference sequence for this species in GenBank (JN810534.1), so it is highly likely that this discrepancy is due to sequencing errors in the original sample or to incorrect taxonomic identification of the reference specimen. Consequently, this highlights the critical need for expanding the reference database; incorporating a larger number of sequences from diverse specimens would significantly enhance diagnostic security, provide greater statistical confidence, and ensure more robust taxonomic discrimination within the genus. This is further reinforced by the fact that the ND2 marker has consistently obtained identity values above 98% for the vast majority of attributions made to other species.

To ensure maximum taxonomic accuracy, we established a strict identification framework based on the prior validation of our molecular markers for each specific group. Based on the prior validation of the molecular markers, it was found that both *CYTB* and *ND2* were effective in identifying *Sporophila lineola* and *S. plumbea*. For the species *S. bouvreuil* and *S. leucoptera*, the *CYTB* gene demonstrated greater potential for molecular discrimination. In contrast, neither marker proved reliable for resolving *S. collaris*, *S. caerulescens*, or *S. nigricollis*, and they were excluded from high-confidence assignments. Therefore, by selectively analyzing only the female specimens that belonged to species with previously proven discrimination efficiency, it is concluded that 52.13% (49 sequences) of the total female sequences in our dataset could be identified with high confidence. This percentage reflects the exact proportion of samples that could be safely resolved using our strictly validated marker-species combinations.

The relatively limited success in species identification for female individuals highlights that standard single-locus genetic barcodes may not always provide sufficient resolution for reliable species assignment in closely related groups. Nevertheless, standard DNA barcoding remains a highly cost-effective and accessible methodology; if these markers successfully discriminate the targeted species under study, they represent the ideal primary tool, eliminating the immediate need for complex and expensive high-throughput sequencing. To overcome limitations only when these cost-effective barcodes fail, alternative and more costly molecular approaches must be considered, such as whole genome sequencing. Alternative, higher-cost methodologies, such as whole-genome sequencing, are justified strictly for identifying diagnostic nucleotide differences that allow for the design of targeted primers; these primers would be capable of amplifying the region that differentiates the species under study and could subsequently be utilized due to the specific and informative features of these regions.

Beyond these methodological balances between cost and genomic resolution, complete diagnostic reliability also strongly depends on the completeness of the database. Finally, a potential limitation of the present study is the lack of comprehensive geographic sampling across the entire distribution area of the analyzed *Sporophila* species. Many seedeaters exhibit wide geographic distribution, which can lead to intraspecific genetic variation and phylogeographic structuring. As our dataset may not fully capture this geographic diversity, future studies incorporating samples from a wider geographic area are needed to assess how intraspecific variation impacts this. Despite these future possibilities, this study already represents a crucial advance in molecular biology, providing an immediate, accessible, and powerful tool to directly assist in law enforcement and the protection of these songbirds, which are highly targeted by traffickers, and in aiding the conservation of these species.

## Conclusion

This study examined the effectiveness of species-specific molecular markers including those developed for *Sporophila* for *CYTB* and *ND2* gene, and the *COI* marker selected from the literature, in distinguishing species within the genus *Sporophila*. Findings indicate that molecular markers from the *CYTB*, *COI*, and *ND2* genes, along with concatenated sequences, effectively distinguish *S. americana*, *S. angolensis*, *S. lineola*, *S. maximiliani*, and *S. plumbea*. The *CYTB* gene was the most suitable marker for *S. bouvreuil*, while the *COI* marker was more effective for *S. albogularis*. For *S. leucoptera*, *CYTB*, *COI*, and concatenated sequences were all effective. However, no reliable markers are currently available to distinguish *S. caerulescens*, *S. collaris*, or *S. nigricollis*. Due to the small sample size, no definitive conclusions could be drawn for *S. frontalis* and *S. hypoxantha*, highlighting the need for further research on these species. Ultimately, this research demonstrates that DNA barcoding is capable of reliably identifying some of the most targeted *Sporophila* species in the illegal trade. As these specific *Sporophila* species are targeted nationwide, the application of these validated markers can be expanded to other locations, encompassing other rescue facilities and enforcement jurisdictions in different regions. Despite the recognized need to expand geographic sampling in future studies to test robustness against intraspecific variation, this study represents a crucial advance in wildlife protection, providing an immediate and practical framework to directly assist conservation and law enforcement regarding these trafficked birds.

## Supplementary Information

Below is the link to the electronic supplementary material.

Supplementary Material 1

Supplementary Material 2

Supplementary Material 3

Supplementary Material 4

Supplementary Material 5

Supplementary Material 6

Supplementary Material 7

Supplementary Material 8

Supplementary Material 9

Supplementary Material 10

Supplementary Material 11

## Data Availability

Sequences were submitted to Nucleotide NCBI and are available under the following accession numbers: PZ295779–PZ295840 for the COI gene, PZ321016–PZ321124 for the CYTB gene, and PZ321125–PZ321233 for the ND2 gene.

## Abbreviations

BLAST: Basic Local Alignment Search Tool
BOLD: Barcode of Life Data System
CEUA: Comissão de Ética no Uso de Animais
*COI*: *Cytochrome C Oxidase I gene*
CETAS: Centro de Triagem de Animais Silvestres
*CYTB*: Cytochrome B gene
EXO-SAP enzymes: Exonuclease I / Shrimp Alkaline Phosphatase
IUCN: International Union for Conservation of Nature
K2P: Kimura 2-parameter
LGBio: Laboratório de Genética & Biodiversidade
mtDNA: Mitochondrial DNA
NCBI: National Center for Biotechnology Information
*ND2*: *NADH Dehydrogenase 2 *gene
pb: Base pairs
PCR: Polymerase Chain Reaction
Sisgen: Sistema Nacional de Gestão do Patrimônio Genético e do Conhecimento Tradicional associado
Taq: *Thermus aquaticus*
UFG: Universidade Federal de Goiás
