# Supplementary material for: DNA barcode markers for birds of the genus Sporophila: advances in species identification
Source: Mol Biol Rep. 2026 Aug 6;53(1):1347. doi: 10.1007/s11033-026-12530-2 (PMC13447409; doi:10.1007/s11033-026-12530-2)
Supplement: Supplementary file 1 [file 11033_2026_12530_MOESM1_ESM.docx]

### **Molecular Biology Reports**

**Title:** **DNA Barcode Markers for Birds of the Genus *Sporophila*: Advances in Species Identification**

**Authors:** Júllia Costa dos Reis¹^*^, Amanda Alves de Melo-Ximenes¹, Rhewter Nunes^2^, Luiz Alfredo Martins Baptista^3^, Leo Caetano Fernandes Silva^3^, Mariana Pires de Campos Telles^4^

**Affiliations:** ^1^ Laboratório de Genética e Biodiversidade, Universidade Federal de Goiás (LGBio), Instituto de Ciências Biológicas, Goiânia, Goiás, Brazil.

^2^ Laboratório de Bioinformática e Biodiversidade (LBB), Instituto Acadêmico de Ciências da Saúde e Biológicas, Universidade Estadual de Goiás, Campus Oeste, UnU de Iporá, Iporá, Goiás, Brazil.

^3^ Instituto Brasileiro do Meio Ambiente e dos Recursos Naturais Renováveis (Ibama), Goiânia, Goiás, Brasil.

^4^ Escola de Ciências Médicas e da Vida, PUC Goiás.

***Email for correspondence:** [jullia_costa@discente.ufg.br](mailto:jullia_costa@discente.ufg.br)

**Fig. 1** Maximum likelihood phylogenetic tree of the genus *Sporophila* (35 species) reconstructed from a 471-character alignment of the mitochondrial *CYTB* gene. Node values represent bootstrap support values calculated from 1,000 pseudo-replicates. Colors indicate distinct species

**Fig. 2** Phylogenetic relationships among 33 species within the genus *Sporophila* based on *ND2* gene sequences (429 characters), modeled via Maximum Likelihood. Branch numbers depict bootstrap support from 1,000 replicates, and colors differentiate each species

**Fig. 3** Maximum likelihood phylogenetic tree of the genus *Sporophila* (29 species) reconstructed from a 424-character alignment of the mitochondrial *COI* gene. Node values represent bootstrap support values calculated from 1,000 pseudo-replicates. Colors indicate distinct species

**Fig. 4** Haplotype network of the mitochondrial *CYTB* gene (457 bp) reconstructed for 62 adult male individuals across 13 *Sporophila* species. Circular nodes represent distinct haplotypes. Species assignments are color-coded. Linear marks on the lines connecting haplotypes represent substituitions separating them

**Fig. 5** Haplotype network of the mitochondrial *ND2* gene (417 bp) reconstructed for 62 adult male individuals across 13 *Sporophila* species. Circular nodes represent distinct haplotypes, scaled in proportion to their absolute frequency within the dataset. Linear marks on the lines connecting haplotypes represent substitutions separating them

**Fig. 6** Haplotype network of the mitochondrial *COI* gene (412 bp) reconstructed for 62 adult male individuals across 13 *Sporophila* species. Circular nodes represent distinct haplotypes, scaled in proportion to their absolute frequency within the dataset. Linear marks on the lines connecting haplotypes represent substitutions separating them
